# A second monoclinic polymorph of 4,4′-[butane-1,4-diylbis(nitrilo­methyl­idyne)]dibenzonitrile

**DOI:** 10.1107/S1600536808037537

**Published:** 2008-11-20

**Authors:** Reza Kia, Hoong-Kun Fun, Hadi Kargar

**Affiliations:** a X-ray Crystallography Unit, School of Physics, Universiti Sains Malaysia, 11800 USM, Penang, Malaysia; b Department of Chemistry, School of Science, Payame Noor University (PNU), Ardakan, Yazd, Iran

## Abstract

The asymmetric unit of the title Schiff base compound, C_20_H_18_N_4_, contains one half-mol­ecule, lying across a crystallographic inversion centre and adopting an *E* configuration with respect to the C=N bonds. The imino group is coplanar with the benzene ring with a maximun deviation of 0.096 (1) Å for the N atom. Within the molecule, the planar units are parallel but extend in opposite directions from the methylene bridge. In the crystal structure, neighbouring mol­ecules are linked together by weak inter­molecular C—H⋯N hydrogen bonds involving the cyano N atoms, forming *R*_2_^2^(10) ring motifs.

## Related literature

For general background, see: Casellato & Vigato (1977 ▶); Calligaris & Randaccio (1987 ▶). For related structures, see: Fun *et al.* (2008 ▶); Fun, Kia & Kargar (2008*a* ▶,*b* ▶); Fun & Kia (2008*a* ▶,*b* ▶). For bond-length data, see: Allen *et al.* (1987 ▶). For hydrogen-bond motifs, see: Bernstein *et al.* (1995 ▶).
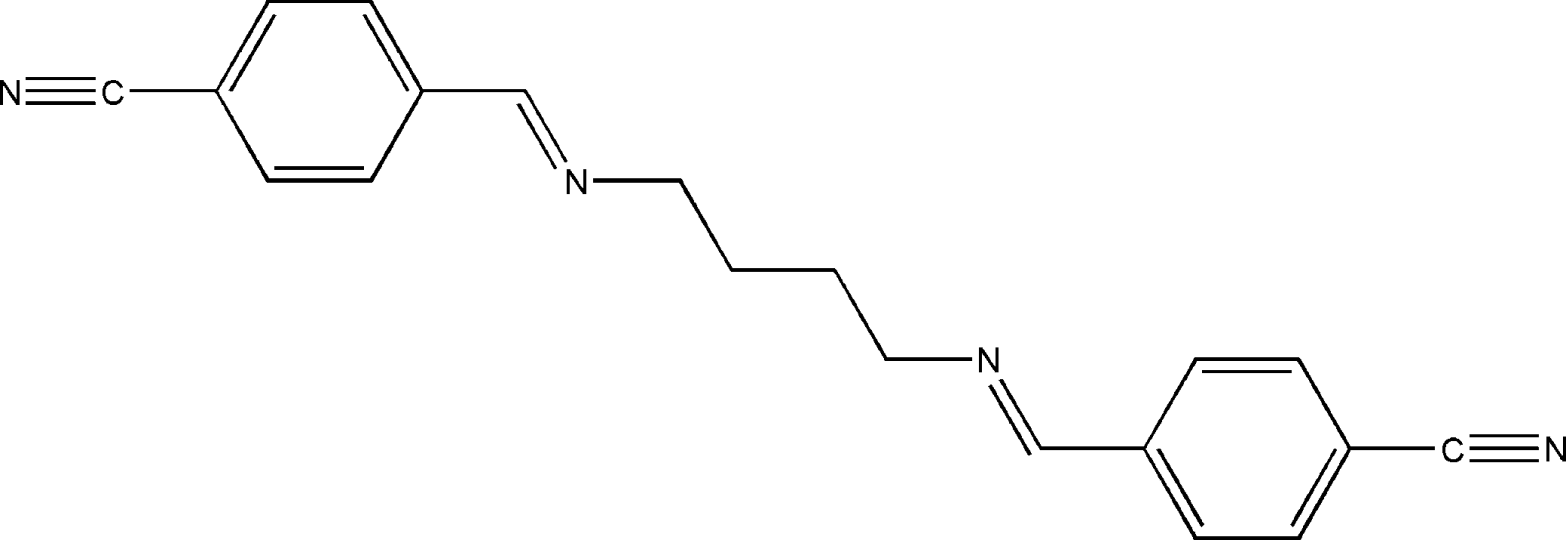


## Experimental

### 

#### Crystal data

C_20_H_18_N_4_
*M* *_r_* = 314.38
Monoclinic, 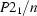

*a* = 4.9958 (1) Å
*b* = 14.8164 (2) Å
*c* = 11.6633 (2) Å
β = 97.310 (1)°
*V* = 856.30 (3) Å^3^
*Z* = 2
Mo *K*α radiation
μ = 0.07 mm^−1^
*T* = 100.0 (1) K
0.39 × 0.29 × 0.28 mm

#### Data collection

Bruker SMART APEXII CCD area-detector diffractometer
Absorption correction: multi-scan (*SADABS*; Bruker, 2005 ▶) *T*_min_ = 0.891, *T*_max_ = 0.979
18411 measured reflections
4473 independent reflections
3659 reflections with *I* > 2σ(*I*)
*R*_int_ = 0.026

#### Refinement

*R*[*F* ^2^ > 2σ(*F* ^2^)] = 0.044
*wR*(*F* ^2^) = 0.135
*S* = 1.04
4473 reflections
109 parameters
H-atom parameters constrained
Δρ_max_ = 0.55 e Å^−3^
Δρ_min_ = −0.26 e Å^−3^

### 

Data collection: *APEX2* (Bruker, 2005 ▶); cell refinement: *SAINT* (Bruker, 2005 ▶); data reduction: *SAINT*; program(s) used to solve structure: *SHELXTL* (Sheldrick, 2008 ▶); program(s) used to refine structure: *SHELXTL*; molecular graphics: *SHELXTL*; software used to prepare material for publication: *SHELXTL* and *PLATON* (Spek, 2003 ▶).

## Supplementary Material

Crystal structure: contains datablocks global, I. DOI: 10.1107/S1600536808037537/hk2569sup1.cif

Structure factors: contains datablocks I. DOI: 10.1107/S1600536808037537/hk2569Isup2.hkl

Additional supplementary materials:  crystallographic information; 3D view; checkCIF report

## Figures and Tables

**Table 1:** Hydrogen-bond geometry (Å, °)

*D*—H⋯*A*	*D*—H	H⋯*A*	*D*⋯*A*	*D*—H⋯*A*
C2—H2*A*⋯N2^i^	0.93	2.52	3.4037 (11)	158

Symmetry code: (i) 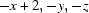.

**Fig. 1.**
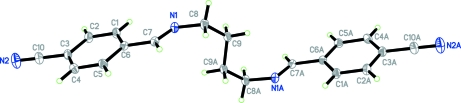
The molecular structure of the title molecule, with the atom-numbering scheme. Displacement ellipsoids are drawn at the 50% probability level [symmetry code: (A) -x, 1 - y, -z].

**Fig. 2.**
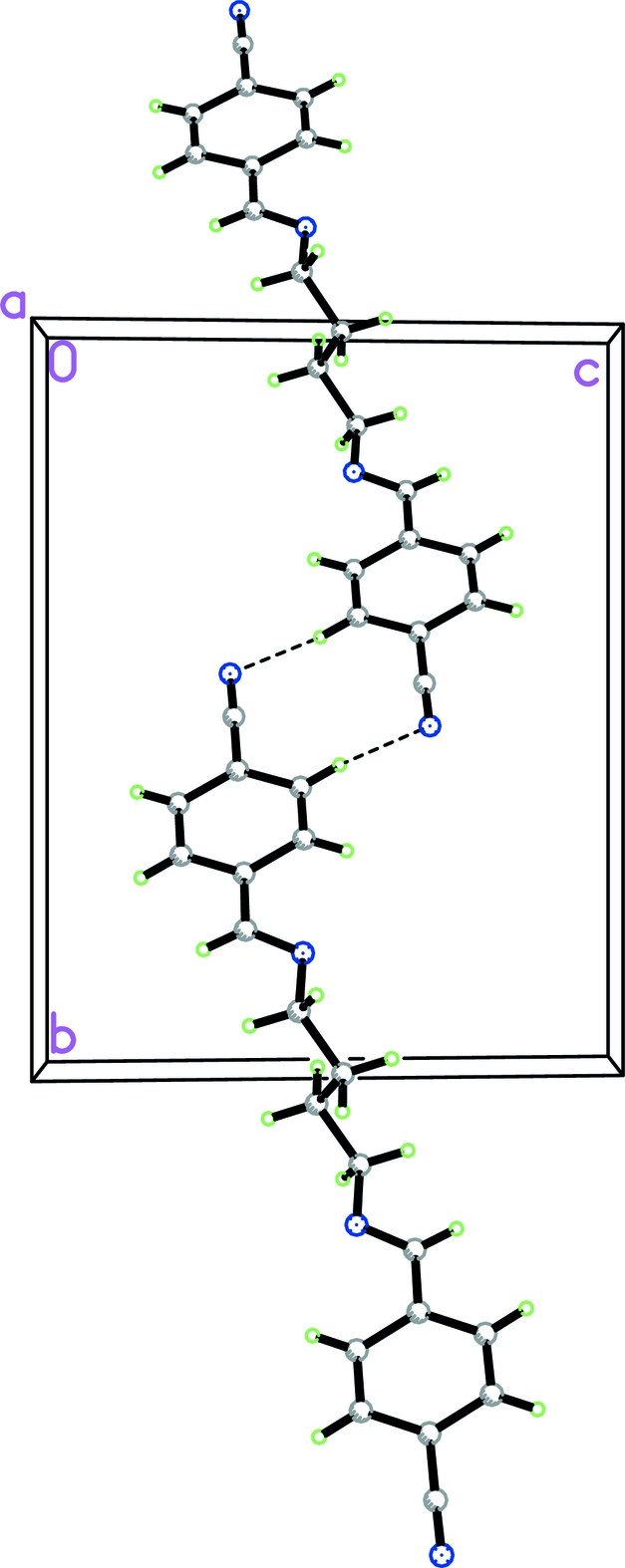
A partial packing diagram viewed down the a axis, showing R22(10) ring motifs. Hydrogen bonds are shown as dashed lines.

## supplementary crystallographic information

### Comment

The condensation of primary amines with carbonyl compounds yields Schiff base compounds (Casellato & Vigato, 1977); these are still one of the most prevalent mixed-donor ligands in coordination chemistry. In the past two decades, the syntheses, structures and properties of Schiff base complexes have stimulated much interest due to their noteworthy contributions in single molecule-based magnetism, materials science and the catalysis of many reactions such as carbonylation, hydroformylation, reduction, oxidation, epoxidation and hydrolysis (Casellato & Vigato 1977). In comparison to the Schiff base metal complexes, only a relatively small number of free Schiff base ligands have been characterized structurally (Calligaris & Randaccio, 1987). As an extension of our work (Fun *et al.*,  2008; Fun, Kia & Kargar 2008a,b; Fun & Kia 2008a,b) on the structural characterization of Schiff base ligands, we reported herein the crystal structure of the title compound.

The asymmetric unit of the title compound contains one-half molecule (Fig. 1), lying across a crystallographic inversion centre and adopting E configurations with respect to the C═N bonds. The bond lengths (Allen *et al.*, 1987) and angles are within normal ranges and are comparable with the related structure (Fun *et al.*,  2008). The imino group is coplanar with the benzene ring, and the planar units are parallel but extend in opposite directions from the methylene bridge.

In the crystal structure, neighbouring molecules are linked together by weak intermolecular C-H···N hydrogen bonds (Table 1) involving the cyano N atoms, forming ten-membered rings with *R*_2_^2^(10) ring motifs (Bernstein *et al.*,  1995).

### Experimental

The synthetic method has been described earlier (Fun, Kia & Kargar, 2008b). Single crystals suitable for X-ray analysis were obtained by evaporation of an ethanol solution at room temperature.

### Refinement

H atoms were positioned geometrically, with C-H = 0.93 and 0.97 Å for aromatic and methylene H, respectively, and constrained to ride on their parent atoms with U_iso_(H) = 1.2U_eq_(C). The highest peak is located 0.68 Å from C5 atom.

### Crystal data

**Table:**

C_20_H_18_N_4_	*F*_000_ = 332
*M**_r_* = 314.38	*D*_x_ = 1.219 Mg m^−^^3^
Monoclinic, *P*2_1_/*n*	Mo *K*α radiation λ = 0.71073 Å
Hall symbol: -P 2yn	Cell parameters from 6822 reflections
*a* = 4.9958 (1) Å	θ = 2.2–39.9º
*b* = 14.8164 (2) Å	µ = 0.08 mm^−^^1^
*c* = 11.6633 (2) Å	*T* = 100.0 (1) K
β = 97.310 (1)º	Block, yellow
*V* = 856.30 (3) Å^3^	0.39 × 0.29 × 0.28 mm
*Z* = 2	

### Data collection

**Table:**

Bruker SMART APEXII CCD area-detector diffractometer	4473 independent reflections
Radiation source: fine-focus sealed tube	3659 reflections with *I* > 2σ(*I*)
Monochromator: graphite	*R*_int_ = 0.026
*T* = 100.0(1) K	θ_max_ = 37.5º
φ and ω scans	θ_min_ = 2.8º
Absorption correction: multi-scan(SADABS; Bruker, 2005)	*h* = −8→8
*T*_min_ = 0.891, *T*_max_ = 0.979	*k* = −24→25
18411 measured reflections	*l* = −19→18

### Refinement

**Table:**

Refinement on *F*^2^	Secondary atom site location: difference Fourier map
Least-squares matrix: full	Hydrogen site location: inferred from neighbouring sites
*R*[*F*^2^ > 2σ(*F*^2^)] = 0.044	H-atom parameters constrained
*wR*(*F*^2^) = 0.135	*w* = 1/[σ^2^(*F*_o_^2^) + (0.0727*P*)^2^ + 0.1307*P*] where *P* = (*F*_o_^2^ + 2*F*_c_^2^)/3
*S* = 1.04	(Δ/σ)_max_ < 0.001
4473 reflections	Δρ_max_ = 0.55 e Å^−^^3^
109 parameters	Δρ_min_ = −0.26 e Å^−^^3^
Primary atom site location: structure-invariant direct methods	Extinction correction: none

### Special details

**Table:**

Experimental. The low-temperature data was collected with the Oxford Cyrosystem Cobra low-temperature attachment.
Geometry. All esds (except the esd in the dihedral angle between two l.s. planes) are estimated using the full covariance matrix. The cell esds are taken into account individually in the estimation of esds in distances, angles and torsion angles; correlations between esds in cell parameters are only used when they are defined by crystal symmetry. An approximate (isotropic) treatment of cell esds is used for estimating esds involving l.s. planes.
Refinement. Refinement of F^2^ against ALL reflections. The weighted R-factor wR and goodness of fit S are based on F^2^, conventional R-factors R are based on F, with F set to zero for negative F^2^. The threshold expression of F^2^ > 2sigma(F^2^) is used only for calculating R-factors(gt) etc. and is not relevant to the choice of reflections for refinement. R-factors based on F^2^ are statistically about twice as large as those based on F, and R- factors based on ALL data will be even larger.

### Fractional atomic coordinates and isotropic or equivalent isotropic displacement parameters (Å^2^)

**Table:**

	*x*	*y*	*z*	*U*_iso_*/*U*_eq_	
N1	0.01080 (11)	0.32548 (4)	0.04403 (5)	0.01925 (11)	
N2	1.09394 (17)	−0.03532 (5)	0.17351 (7)	0.03381 (17)	
C1	0.40142 (14)	0.18220 (4)	0.04338 (6)	0.01953 (12)	
H1A	0.3066	0.1973	−0.0279	0.023*	
C2	0.59829 (14)	0.11593 (5)	0.04968 (6)	0.02080 (12)	
H2A	0.6360	0.0866	−0.0169	0.025*	
C3	0.74003 (13)	0.09356 (4)	0.15748 (6)	0.01898 (11)	
C4	0.68642 (13)	0.13779 (5)	0.25771 (6)	0.02032 (12)	
H4A	0.7827	0.1231	0.3288	0.024*	
C5	0.48772 (13)	0.20402 (4)	0.25021 (5)	0.01888 (11)	
H5A	0.4503	0.2335	0.3168	0.023*	
C6	0.34401 (12)	0.22663 (4)	0.14350 (5)	0.01610 (11)	
C7	0.13465 (12)	0.29707 (4)	0.13917 (5)	0.01716 (11)	
H7A	0.0919	0.3215	0.2080	0.021*	
C8	−0.19246 (13)	0.39541 (4)	0.05029 (6)	0.02132 (12)	
H8A	−0.1937	0.4135	0.1301	0.026*	
H8B	−0.3694	0.3715	0.0221	0.026*	
C9	−0.13397 (12)	0.47738 (4)	−0.02174 (6)	0.01933 (12)	
H9A	−0.1314	0.4587	−0.1013	0.023*	
H9B	−0.2785	0.5209	−0.0204	0.023*	
C10	0.93805 (15)	0.02262 (5)	0.16554 (6)	0.02439 (14)	

### Atomic displacement parameters (Å^2^)

**Table:**

	*U*^11^	*U*^22^	*U*^33^	*U*^12^	*U*^13^	*U*^23^
N1	0.0200 (2)	0.0161 (2)	0.0217 (2)	0.00380 (17)	0.00313 (18)	0.00211 (17)
N2	0.0380 (4)	0.0346 (4)	0.0290 (3)	0.0179 (3)	0.0052 (3)	0.0058 (3)
C1	0.0237 (3)	0.0179 (2)	0.0164 (2)	0.0047 (2)	0.00064 (19)	0.00065 (19)
C2	0.0249 (3)	0.0188 (3)	0.0187 (3)	0.0055 (2)	0.0026 (2)	0.0003 (2)
C3	0.0188 (2)	0.0166 (2)	0.0215 (3)	0.00276 (18)	0.00223 (19)	0.00326 (19)
C4	0.0197 (2)	0.0220 (3)	0.0185 (3)	0.0020 (2)	−0.00060 (19)	0.0026 (2)
C5	0.0199 (2)	0.0200 (3)	0.0163 (2)	0.00113 (19)	0.00059 (18)	−0.00036 (19)
C6	0.0173 (2)	0.0140 (2)	0.0168 (2)	0.00002 (17)	0.00157 (17)	0.00093 (17)
C7	0.0183 (2)	0.0148 (2)	0.0187 (2)	0.00041 (17)	0.00331 (18)	−0.00005 (18)
C8	0.0179 (2)	0.0180 (2)	0.0289 (3)	0.00373 (19)	0.0065 (2)	0.0041 (2)
C9	0.0152 (2)	0.0177 (2)	0.0252 (3)	0.00334 (17)	0.00291 (19)	0.0039 (2)
C10	0.0252 (3)	0.0242 (3)	0.0238 (3)	0.0065 (2)	0.0032 (2)	0.0043 (2)

### Geometric parameters (Å, °)

**Table:**

N1—C7	1.2714 (8)	C4—H4A	0.9300
N1—C8	1.4590 (8)	C5—C6	1.3962 (9)
N2—C10	1.1548 (9)	C5—H5A	0.9300
C1—C2	1.3851 (9)	C6—C7	1.4740 (8)
C1—C6	1.4015 (9)	C7—H7A	0.9300
C1—H1A	0.9300	C8—C9	1.5260 (9)
C2—C3	1.4019 (9)	C8—H8A	0.9700
C2—H2A	0.9300	C8—H8B	0.9700
C3—C4	1.3955 (10)	C9—C9^i^	1.5249 (13)
C3—C10	1.4382 (9)	C9—H9A	0.9700
C4—C5	1.3906 (9)	C9—H9B	0.9700
			
C7—N1—C8	117.13 (6)	C1—C6—C7	121.58 (5)
C2—C1—C6	120.53 (6)	N1—C7—C6	121.92 (6)
C2—C1—H1A	119.7	N1—C7—H7A	119.0
C6—C1—H1A	119.7	C6—C7—H7A	119.0
C1—C2—C3	119.30 (6)	N1—C8—C9	110.76 (5)
C1—C2—H2A	120.3	N1—C8—H8A	109.5
C3—C2—H2A	120.3	C9—C8—H8A	109.5
C4—C3—C2	120.75 (6)	N1—C8—H8B	109.5
C4—C3—C10	119.48 (6)	C9—C8—H8B	109.5
C2—C3—C10	119.75 (6)	H8A—C8—H8B	108.1
C5—C4—C3	119.36 (6)	C9^i^—C9—C8	112.85 (7)
C5—C4—H4A	120.3	C9^i^—C9—H9A	109.0
C3—C4—H4A	120.3	C8—C9—H9A	109.0
C4—C5—C6	120.49 (6)	C9^i^—C9—H9B	109.0
C4—C5—H5A	119.8	C8—C9—H9B	109.0
C6—C5—H5A	119.8	H9A—C9—H9B	107.8
C5—C6—C1	119.57 (6)	N2—C10—C3	178.58 (8)
C5—C6—C7	118.86 (5)		
			
C6—C1—C2—C3	0.04 (10)	C2—C1—C6—C5	0.30 (10)
C1—C2—C3—C4	−0.57 (10)	C2—C1—C6—C7	−179.71 (6)
C1—C2—C3—C10	177.78 (6)	C8—N1—C7—C6	−179.98 (5)
C2—C3—C4—C5	0.76 (10)	C5—C6—C7—N1	174.67 (6)
C10—C3—C4—C5	−177.60 (6)	C1—C6—C7—N1	−5.31 (10)
C3—C4—C5—C6	−0.41 (10)	C7—N1—C8—C9	124.91 (7)
C4—C5—C6—C1	−0.12 (10)	N1—C8—C9—C9^i^	−62.65 (9)
C4—C5—C6—C7	179.90 (6)		

Symmetry codes: (i) −*x*, −*y*+1, −*z*.

### Hydrogen-bond geometry (Å, °)

**Table:**

*D*—H···*A*	*D*—H	H···*A*	*D*···*A*	*D*—H···*A*
C2—H2A···N2^ii^	0.93	2.52	3.4037 (11)	158

Symmetry codes: (ii) −*x*+2, −*y*, −*z*.
